# The plasma bioavailability of nitrate and betanin from *Beta vulgaris rubra* in humans

**DOI:** 10.1007/s00394-016-1173-5

**Published:** 2016-02-12

**Authors:** Tom Clifford, Costas M. Constantinou, Karen M. Keane, Daniel J. West, Glyn Howatson, Emma J. Stevenson

**Affiliations:** 1 Department of Sport, Exercise and Rehabilitation, Faculty of Health and Life Sciences, Northumbria University, Newcastle-upon-Tyne, NE1 8ST UK; 2 Institute of Cellular Medicine, Newcastle University, Newcastle-upon-Tyne, UK; 3 Water Research Group, School of Environmental Sciences and Development, Northwest University, Potchefstroom, South Africa

**Keywords:** Beetroot, Betalains, Nitric oxide, Antioxidant, Bioavailability

## Abstract

**Purpose:**

To evaluate the plasma bioavailability of betanin and nitric oxide (NOx) after consuming beetroot juice (BTJ) and whole beetroot (BF). BTJ and BF were also analysed for antioxidant capacity, polyphenol content (TPC) and betalain content.

**Methods:**

Ten healthy males consumed either 250 ml of BTJ, 300 g of BF or a placebo drink, in a randomised, crossover design. Venous plasma samples were collected pre (baseline), 1, 2, 3, 5 and 8 h post-ingestion. Betanin content in BTJ, BF and plasma was analysed with reverse-phase high-performance liquid chromatography (HPLC) and mass spectrometry detection (LCMS). Antioxidant capacity was estimated using the Trolox equivalent antioxidant capacity (TEAC) and polyphenol content using Folin–Ciocalteu colorimetric methods [gallic acid equivalents (GAE)] and betalain content spectrophotometrically.

**Results:**

TEAC was 11.4 ± 0.2 mmol/L for BTJ and 3.4 ± 0.4 μmol/g for BF. Both BTJ and BF contained a number of polyphenols (1606.9 ± 151 mg/GAE/L and 1.67 ± 0.1 mg/GAE/g, respectively), betacyanins (68.2 ± 0.4 mg/betanin equivalents/L and 19.6 ± 0.6 mg/betanin equivalents/100 g, respectively) and betaxanthins (41.7 ± 0.7 mg/indicaxanthin equivalents/L and 7.5 ± 0.2 mg/indicaxanthin equivalents/100 g, respectively). Despite high betanin contents in both BTJ (~194 mg) and BF (~66 mg), betanin could not be detected in the plasma at any time point post-ingestion. Plasma NOx was elevated above baseline for 8 h after consuming BTJ and 5 h after BF (*P* < 0.05).

**Conclusions:**

These data reveal that BTJ and BF are rich in phytonutrients and may provide a useful means of increasing plasma NOx bioavailability. However, betanin, the major betalain in beetroot, showed poor bioavailability in plasma.

## Introduction

The root vegetable, red beetroot (*Beta vulgaris rubra*), is a functional food that has attracted much attention over the last decade, with particular focus on its potential health benefits (for review, see [1, 2]). The interest in beetroot has been largely driven by its nitrate content, which is proposed to be ~1459 mg kg^−1^ DW [3]. Dietary nitrate may confer beneficial health effects via its sequential reduction to nitrite and nitric oxide (NOx), a pleiotropic molecule that plays a key role in the regulation of vascular homoeostasis, immune function and metabolism [4, 5]. There are now several reports that acute consumption of beetroot can stimulate endogenous NOx production and evoke positive changes in endothelial function and blood pressure [6–8]. Consequently, beetroot is currently purported as a health promoting food that might be useful for reducing the risk of developing cardiovascular diseases (i.e. hypertension and stroke) and immune disorders (i.e. inflammatory bowel disease) [2, 3, 9].

**Fig. 1:**
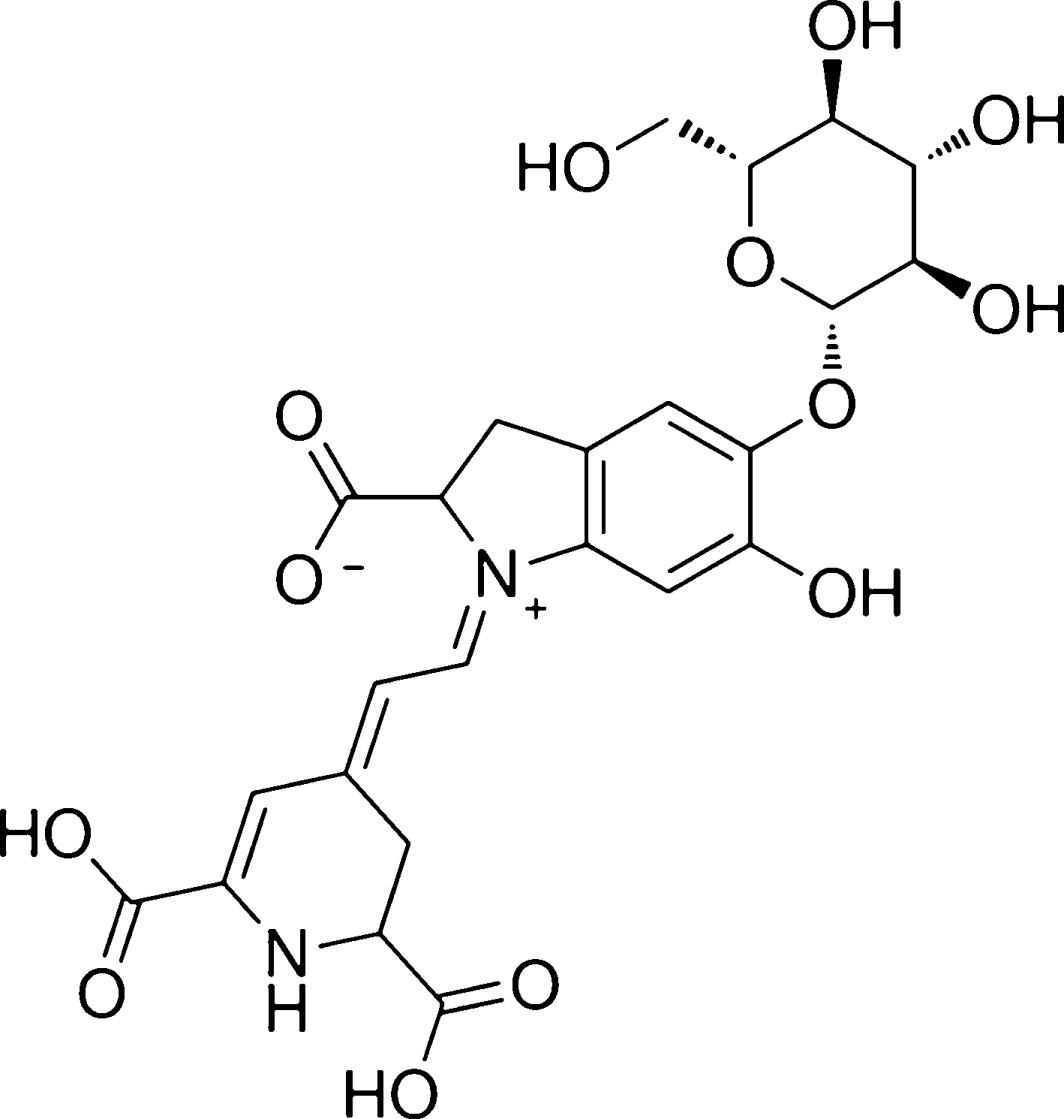
Chemical structure of betanin (betanidin 5-O-b-glucoside)

Nitrate is not the only constituent of beetroot that may have beneficial effects for health. As well as being a good source of polyphenols, beetroot contains a group of betalamic acid derivatives known as betalains [10]. The betalains derive from the plant order *Caryophyllales* and are categorised as either betacyanins, which are responsible for the red/violet colour of red beetroot or betaxanthins, which are yellow in colour [1, 11]. Betalains are water-soluble phytochemicals that have been shown to possess anti-inflammatory, antioxidant and chemo-preventive activities in vitro [1, 10, 12, 13]. In recent years, there has been a particular interest in the biochemical activity of betanin (betanidin 5-O-b-glucoside; chemical structure depicted by Fig. 1), which is the most abundant betacyanin in beetroot (300–600 mg/kg uncooked), and the main constituent of the food colourant E162 [10, 14–16]. As a cationised compound, betanin is a highly effective scavenger of reactive oxygen species (ROS) [14, 17]. Betanin also possesses anti-inflammatory properties; betanin and its aglycone betanidin were shown to modulate lipoxygenase and cyclooxygenase activity in vitro, indicating that betanin might downregulate pro-inflammatory signalling [13]. These findings have led to interest in the role of beetroot in the protection against the potentially damaging effects of ROS and aberrant immune function [18, 19].

An increased awareness of beetroot’s potential health benefits, together with an increased demand for convenient health foods, has led to the development of a number of beetroot-based functional food products. Throughout the world, beetroot can now be purchased as a concentrated juice drink or as a ready to consume precooked snack, bypassing the need for prior cooking. Given the wide array of bioactive compounds present in beetroot, it is possible that regular consumption of these products could have favourable effects for health and well-being. Furthermore, these products might have applications as dietary supplements in the management of a host of chronic and degenerative disorders, including hypertension, osteoarthritis and numerous cancers [1, 9, 20]. However, in order to evaluate the potential usefulness of these products in the promotion of general health or as therapeutic agents, information on their in vivo bioavailability and phytochemical content in humans is required.

Dietary nitrate is believed to be highly bioavailable [21], and increases in the plasma have been observed following ingestion of beetroot juice [8, 22]. Betalains, in contrast, are thought to have much lower bioavailability [23]; however, no published studies have characterised the bioavailability of betalains in plasma from consuming commercially available beetroot products. Collectively, these data would provide novel and readily accessible information to both consumers and practitioners (i.e. nutritionists, dieticians, physiologists and other health professionals) who may be interested in the potential health benefits of commercially available functional foods, like beetroot.

Consequently, the main objective of this study was to determine the acute plasma bioavailability of betanin, the major betalain in beetroot, and nitrate, a precursor for NOx activity, in human plasma after consuming both beetroot juice (BTJ) and beetroot whole food (BF). A secondary aim was to characterise the antioxidant capacity, polyphenol and betalain content of BTJ and BF.

## Methods

### Participants

Ten healthy, non-smoking males (age 23 ± 3 years; height 1.82 ± 0.60 m; mass 78.8 ± 6.7 kg) were recruited to participate in this study. Participants were excluded from the study if they had any known food allergies, were taking medication (including dietary supplements), or were suffering from or have previous history of renal, gastrointestinal or cardiovascular complications or any other contraindication to the study procedures. All participants were made aware that their participation was voluntary and that they were free to withdraw at any time. The study protocol received institutional ethical approval. Each participant provided written informed consent prior to study entry.

### Experimental design

**Fig. 2:**
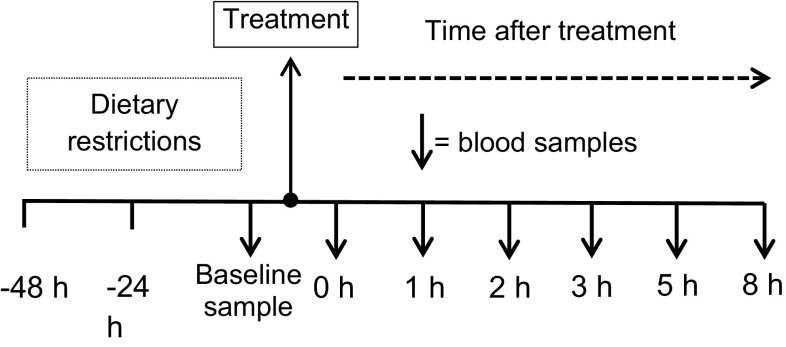
Schematic outline of bioavailability study procedures

Subjects were required to attend the laboratory on 3 occasions, separated by 7 days. In the 48 h prior to each visit, subjects followed a low phenolic and betalanic diet. This included avoiding all vegetables, cured meats, fruits and their juices, chocolate, wholegrain breads and grains, caffeinated beverages including all varieties of tea, coffee and alcohol. To ensure compliance, subjects were given a written list of foods to avoid and diaries to record their intake 48 h prior to the first trial. They were instructed to replicate this diet as closely as possible in the 48 h before the remaining two trials. For each trial, subjects attended the laboratory between the hours of 07:00–09:00 following a 12-h overnight fast and had a cannula inserted into a vein at the antecubital fossa. After a baseline blood sample, subjects were given 1 of 3 treatments; beetroot juice (250 ml), an isocaloric placebo (250 ml) or cooked beetroot (300 g) in a randomised, crossover fashion (see Table 1). Further blood samples were drawn at 1, 2, 3, 5 and 8 h after ingesting the treatments (see Fig. 2 for schematic) to ascertain the pharmacokinetics of the compounds of interest. Subjects were not allowed to consume any food until testing was complete but were allowed water ad libitum; the amount of water consumed on the first trial was recorded and replicated in the subsequent trials.

**Table 1:** Nutrient composition of treatment beverages

Treatment	BTJ	BF	PLA
Energy (Kcals)	81	126	76.8
Volume	250 ml	300 g	250 ml
Carbohydrate (g)	16.4	23.1	16.4
Protein (g)	2.8	3.6	2.8
Fat (g)	0.4	0.6	Trace
Nitrate (mg)	~250.6 mg	N/A	Trace

### Blood sampling procedures

At each time point, 10 ml of venous blood was collected into EDTA treated tubes (Vacutainer, Bendict Dickinson) and immediately centrifuged at 3000*g* (4°) for 10 min to separate plasma. Samples were aspirated into a series of aliquots and stored immediately at −80 °C until analysis.

### Supplementation

Nutritional composition of the 3 treatments is provided in Table 1. The beetroot juice (Love Beets Super Tasty Beetroot Juice, Gs Fresh Ltd., Cambridgeshire, UK) and the whole beetroot food were freshly cooked and pre-packed (Tesco PLC, Hertfordshire, UK). Both the beetroot juice and beetroot food originated from the same manufacturer (Gs Fresh Ltd., Cambridgeshire, UK). The placebo (PLA) beverage contained a commercially available fruit <1 % squash (Kia Ora, Coca Cola Enterprises, Uxbridge, UK) with negligible phytochemical and nitrate content and was fortified with maltodextrin (Myprotein, Manchester, UK) flavourless protein powder (Arla Foods, Amba, Denmark) and water, to match the BTJ for volume and macro-nutrient content. The quantification of nitrate in the BTJ was performed by Gs Fresh Ltd; however, data on the nitrate content of BF were not available.

### Antioxidant activity and phenolic content

Extracts of the whole beetroot samples in 80:20 methanol:water, and mixtures of placebo and beetroot juice in the same solvent were used to determine their total phenolic content (TPC) and antioxidant activity (TEAC). For the beetroot food, samples (about 10 g of whole beetroot) were cryogenically milled in liquid nitrogen using an IKA A11 S2 analytical mill (IKA Works, Wilmington, USA). Accurate amounts of frozen powder (500–700 mg) were extracted five times with 1 mL 80:20 methanol:water, filtered and combined to a total volume of 5 mL, whilst the liquid materials (juice and placebo) were diluted (1:10) in the extraction solvent.

A modified 2,2-diphenyl-1-picrylhydrazyl (DPPH) assay used for antioxidant activity measurements was adjusted for use in the present study [24]. The DPPH solution was prepared freshly before the analysis, by dissolving the DPPH reagent (2.4 mg) in 80 % methanol (100 mL). Samples were further diluted in deionised water (1:10 or 1:100) and 10 µL of sample; 40 µL of deionised water and 200 µL of DPPH solution were added into each well of the CELLSTAR 96 well plate (Greiner Bio-One, Monroe, USA). Absorbance readings were taken at 515 nm, at 3-min intervals over a 30-min period at 37 °C, using a BioTek Synergy HT Multi-Mode Microplate Reader (BioTek, Winooski, USA). A calibration curve using Trolox (0–500 µM, *R* ^2^ = 0.99) was plotted. Final values are expressed as means of Trolox equivalents per milligram of sample ± SD for 6 replicants.

Total phenolic content (TPC) was measured using a modified Folin–Ciocalteu colourimetric method [25, 26]. Samples were diluted in deionised water (1:10 or 1:100) and 50 µL of the diluted extract; 50 µL of Folin–Ciocalteu reagent diluted in water (1:25) and 100 µL of 6 % (w/v) sodium carbonate were added into corresponding sample wells of a 96-well plate (Greiner Bio-One, Monroe, USA). Absorbance readings were taken at 725 nm, at 5-min intervals, over a 30-min period at 25 °C using a BioTek Synergy HT Multi-Mode Microplate Reader (BioTek, Winooski, USA). A stock solution of gallic acid (5.9 mM) was prepared in aqueous methanol (80 % (v/v), and quantification was performed on the basis of a standard curve in the range 0–50 mg/mL (*R* ^2^ = 0.99). The analysed samples were measured versus a blank sample. All values are expressed as means of gallic acid equivalents per gram of sample ± SD for 6 replicants.

### Total betanin and betaxanthin content

The content of betaxanthins and betacyanins in the buffered extract and 1:10 juice solutions in aqueous McIlvaine buffer (pH 6.5) was determined at 538 nm and 480 nm with a UV–Vis spectrometer (Ultrospec 2000 UV/Vis spectrophotometer, Pharmacia Biotech, Sweden), respectively, according to the methods of Cai and Corke [27] and Mossamer et al. [28]. Total betalains were quantified using the following equation: BLC [mg/L] = (*A* × DF × MW × 1000)/(*ε* × 1), where *A* is the absorption value at the absorption maximum, DF the dilution factor and 1 the path length (1 cm) of the cuvette. For quantification of betacyanins (Bc) and betaxanthins (Bx), the molecular weights (MW) and molar extinction coefficients (*ε*) of betanin (MW = 550 g/mol; *ε* = 60,000 L/mol cm; *λ* = 538 nm) and indicaxanthin (MW = 308 g/mol; *ε* = 48,000 L/mol cm; *λ* = 480 nm) were applied, respectively.

### Extraction of whole beetroot betalains

A previously described method [29, 30] was used for betalain extraction of whole beetroots. Samples (about 10 g of whole beetroot) were cryogenically milled in liquid nitrogen using an IKA A11 S2 analytical mill (IKA Works, Wilmington, USA). Accurate amounts of frozen powder (500–600 mg) were transferred in a 15-mL Falcon tube and homogenised with 1 mL water for 5 min (Whirlimixer, FisherBrand, Fisher Scientific, UK). The homogenate was centrifuged for 15 min at 3000 rpm, and the supernatant was collected. The insoluble part was re-extracted with 1 mL of water for a total of five times. The extracts were combined, and the water was removed using a rotary evaporator, coupled to a heated water bath under vacuum (STUART, Bibby Scientific, Staffordshire, UK) and re-dissolved in McIlvaine buffer (pH 6.5, 10 mL).

### Extraction of beetroot food for betanin determination

Betanin standard was purchased from Adooq Bioscience (California, USA). Beetroot samples (about 20 g of whole beetroot) were cryogenically milled in liquid nitrogen using an IKA A11 S2 analytical mill (IKA Works, Wilmington, USA). Accurate amounts of frozen powder (200–300 mg) were transferred in a 15-mL Falcon tube and homogenised with 1 mL water for 5 min (Whirlimixer, FisherBrand, Fisher Scientific, UK). The homogenate was centrifuged for 15 min at 3000 rpm, and the supernatant was collected. The insoluble part was re-extracted with 1 mL of water for a total of five times. The extracts were combined, and the water was removed using a rotary evaporator, coupled to a heated water bath under vacuum (STUART, Bibby Scientific, Staffordshire, UK). Finally, the extracts were re-dissolved in water (10 mL), filtered and diluted further prior to LC–MS analysis (1:100 in mobile phase A). All experiments were performed in sixplicate.

### LCMS for betanin determination

Betanin determination of diluted beetroot juice samples (2.5 mg/mL in 1:1 0.1 % formic acid in water: 2 % HCl in MeOH) and beetroot food extracts was carried out on a Dionex UltiMate 3000 RSLC HPLC System (Dionex, Camberley, UK) equipped with an UltiMate 3000 RS pump, an UltiMate 3000 RS autosampler and a QExactive Quadrupole-Orbitrap Mass Spectrometer (Thermo Fisher Scientific, Waltham, USA). Electrospray ionisation at negative ion mode was performed with a spray voltage of 2.00 kV and capillary temperature of 280 °C. The total ion current (TIC) with a range of 100–1000 m/z and 70,000 resolution was measured. The ion m/z 549 was used for quantification of betanin. Sample aliquots (3 µL) were injected on a Phenomenex Luna C18(2) (250 × 2.0 mm, 5um particle size) reverse-phase column thermostatically regulated at 40 °C. The mobile phase consisted of water with 1 % acetic acid (solvent A), and acetonitrile with 1 % acetic acid (solvent B). After a 6-min equilibration with 20 % B, the elution programme was as follows: 0–30 min, 10–100 % B, (0.2 mL/min) followed by a washing stage (100 % B, 30–36 min, 0.2 mL/min) and re-equilibration at the initial conditions for 3 min. Betanin with a retention time of 2.56 min was quantified by external standard determination.

### Extraction of plasma for betanin determination

Several attempts to extract betanin from plasma samples were performed: (a) 1 mL of plasma was mixed with 4 mL oxalic Acid (10 nM) and 0.1 mL HCl (12.6 M) in 15-mL Falcon tubes and centrifuged at 826*g* for 5 min. The supernatant was absorbed on to a primed solid-phase extraction cartridge (Waters Sep-Pak c17 plus short cartridge, 360 mg sorbent per cartridge, 55–105 µm), washed with methanol +0.2 % trifluoroacetic acid (TFA) followed by 2 × 5 mL of water. The sample was eluted with 3 mL of MeOH + 0.2 % TFA, dried under N2 at 45 °C. Samples were then reconstituted in 400 µl of solvent F: 0.1 % formic acid in water: 2 % HCl in methanol and filtered through a 0.2-µm polytetrafluoroethylene filter prior to HPLC and LC–MS analyses; (b) 1 mL of plasma was extracted with 4 mL 1:1 acetonitrile:water for 10 min and centrifuged for 10 min at 3000 rpm. The supernatant was collected, evaporated to dryness, reconstituted in 1:1 acetonitrile: water and filtered in autosampler vials. Three types of plasma samples were analysed using the above two methods as well as with variations, samples obtained from test subjects after consumption of beetroot food, beetroot juice and placebo, at various time points, and analysed using HPLC/UV/Vis/FLD and LCMS methodologies.

### Analysis of plasma NO_x_

Plasma NOx bioavailability was determined from plasma nitrate and nitrite concentrations using a standard assay kit (R&D Systems, Minneapolis, Minnesota). The assay quantifies plasma NOx by measuring total nitrite after nitrate has been enzymatically reduced to nitrite via nitrate reductase.

### Data analysis

All data are presented as mean ± standard deviation (SD). A two-way, repeated-measures analyses of variance (ANOVA) was used to test for between trial differences in plasma NOx concentrations; 3 trials (BTJ vs. BF vs. PLA) by 6 time points (baseline, 1, 2, 3, 5 and 8 h post-ingestion). In the event of a significant interaction effect (trial × time), Fisher LSD post hoc analysis was performed to locate pairwise differences occurred. Statistical significance was set at *P* < 0.05 prior to analyses. All analysis was performed with IBM SPSS statistics 20 for Windows (Surrey, UK).

## Results

Inspection of food diaries indicated that participants complied with the imposed dietary restrictions and that their intakes did not differ between trials. No adverse events were reported with any of the supplements.

### Betanin content and bioavailability

**Fig. 3:**
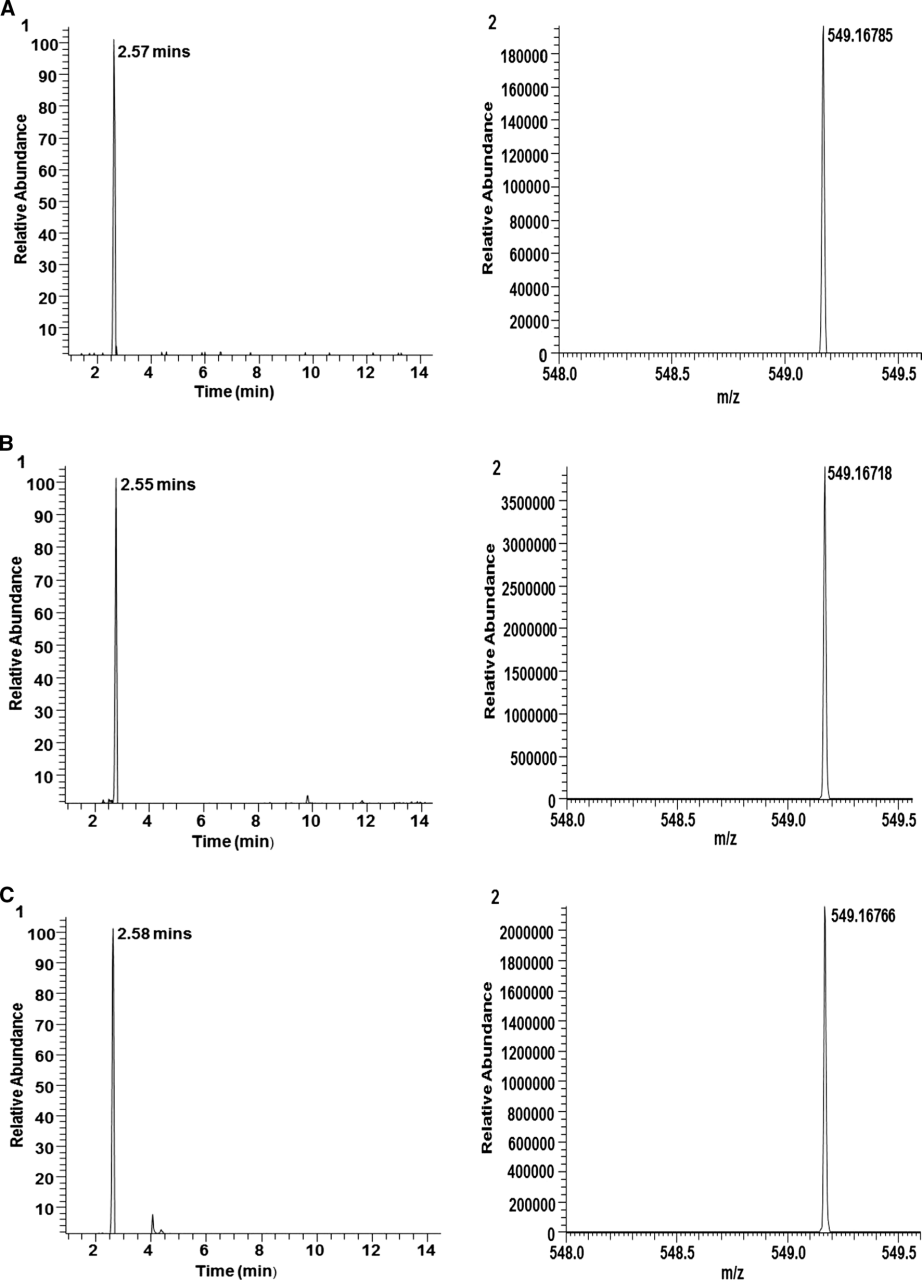
**a** *1* LCMS chromatograms of betanin standard (RT = 2.57 min). (*2*) MS output of betanin standard (base peak m/z 548.5–549.5) **b** (*1*) betanin in BTJ (RT = 2.55). (*2*) MS output for BTJ (base peak m/z 548.5–549.5) **c** (*1*) betanin in BF (RT = 2.56) (*2*) MS output for BF (base peak m/z 548.5–549.5)

Betanin was identified in both the BTJ and BF with LCMS analysis (Fig. 3). Total betanin content for the BTJ and BF is presented in Table 2. Based on these analyses, each bottle of BTJ (250 ml) and serving of BF (300 g) contained ~194 and ~66 mg of betanin, respectively. No betanin was detected in the PLA used in this study. Betanin could also not be detected in the plasma samples obtained after BTJ, BF or PLA consumption (data not shown).

**Table 2:** Trolox equivalence antioxidant activity (TEAC), total polyphenols (TPC), betanin, betacyanin and betaxanthin content of beetroot juice (BTJ), beetroot food (BF) and placebo (PLA)

Treatment	TEAC (mmol/L)	TPC (mg/GAE/L)	Betanin (mg/L)	Total betaxanthins (mg indicaxanthin equivalents/L)	Total betacyanins (mg betanin equivalents/L)
Beetroot juice	11.4 ± 0.2	1606.9 ± 151	777.9 ± 41.3	41.7 ± 0.7	68.2 ± 0.4
Beetroot food	3.4 ± 0.4	1.7 ± 0.1	221.3 ± 23	7.5 ± 0.2	19.6 ± 0.6
Placebo	0.25 ± 0.02	172.3 ± 13.3	ND	ND	ND

Values are mean ± SD

*TEAC* BTJ mmol/L, *BF* μmol/g, *TPC* BTJ, mg gallic acid equivalent/L, *BF* mg gallic acid equivalent/g, *Betanin* BTJ, mg/L, *BF* mg/kg, *Total betaxanthins* BTJ, mg indicaxanthin equivalents/L, *BF* mg indicaxanthin equivalents/100 g fresh weight, *Total betacyanins* BTJ, mg betanin equivalents/L, *BF* mg betanin equivalents/100 g fresh weight

### Composition of beetroot juice and whole beetroot food

Antioxidant capacity, phenolic content, total betalain and total betaxanthin content for the BTJ, BF and PLA are presented in Table 2. According to these data, each serving of beetroot juice (250 ml) had a TEAC of ~3 mmol/l and contained ~405 mg GAE equivalents of phenolic compounds, ~17 mg of betacyanins and ~10 mg of betaxanthins. The analysis of the BF showed that the 300 g serving of BF fed to participants had a TEAC of ~1.01 mmol/L, contained ~501 mg of GAE of phenols, ~59 mg of betacyanins and ~22.5 mg of betaxanthins. The PLA contained a small number of phenolic compounds (~43 mg); however, betalains could not be detected and the TEAC was low (<0.5 mmol/L).

### Plasma NOx concentrations

**Fig. 4:**
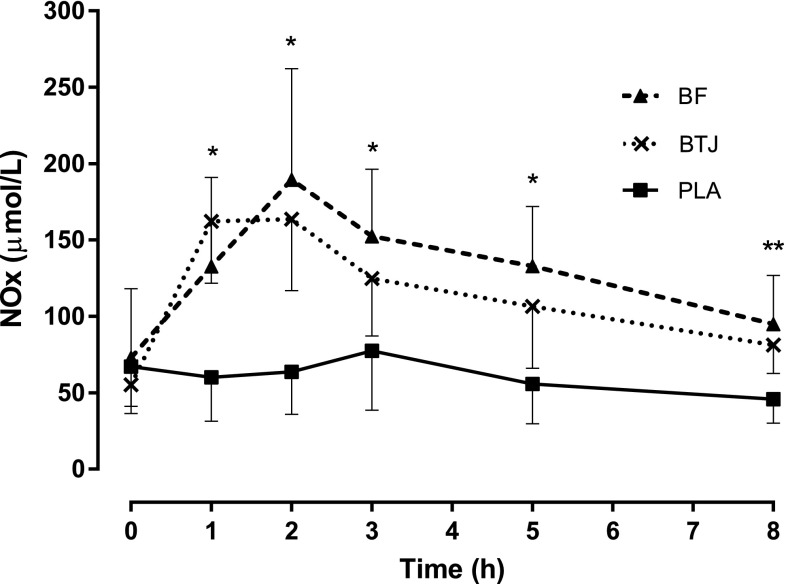
Plasma NOx concentrations after beetroot juice (BTJ), beetroot food (BF) and placebo (PLA) ingestion. Data are mean ± SD; *BTJ and BF higher than PLA (*P* < 0.05), **BTJ higher than PLA (*P* < 0.05)

Data are presented in Fig. 4. At baseline (0 h), concentrations of plasma NOx were similar between trials (*P* > 0.05). In the PLA trial, there was no change in plasma NOx concentrations at any time point (*P* > 0.05); however, after ingestion of both BTJ and BF there was an increase in plasma NOx compared to baseline (time effects; *P* < 0.001) and PLA (drink x time interaction; *P* < 0.001). Plasma NOx reached peak concentrations 2 h post-ingestion in both the BTJ and BF groups (*P* < 0.001; 163.7 ± 46.9 and 189.4 ± 72.8 μmol/L, respectively) and were still elevated above baseline values at 8 h post with BTJ (*P* < 0.001) and 5 h post with BF (*P* = 0.012) (Fig. 4).

## Discussion

The present study aimed to determine the plasma bioavailability of nitrate and betanin after consuming a commercially available BTJ and BF. Both the BTJ and BF were rich in betalains, particularly betanin; however, betanin could not be detected in plasma following consumption. Conversely, and in line with other research, ingestion of BTJ and BF evoked elevations in plasma NOx levels compared to a placebo. Both BTJ and BF were found to contain significant amounts of polyphenols and antioxidant compounds. The present findings provide new information regarding the bioavailability and phytochemical content of two commonly consumed beetroot products.

Antioxidant capacity of the BTJ, BF and PLA was measured using the TEAC assay. Although this was not a primary aim of this study, we felt that this information would be useful for comparisons with other antioxidant-rich foods. The TEAC assay estimates AC by comparing the intervention’s scavenging ability to the Trolox standard [31] and is commonly used to provide an index of a food or beverage antioxidant potential [31, 32]. The analysis revealed the TEAC for BTJ (~11.4 mmol/L) to be higher than values reported for iced tea, green tea, apple juice, cranberry juice and orange juice (4–10 mmo/L), but lower than acai juice, black cherry juice, blueberry juice and pomegranate juice (12–40 mmol/L) (Seeram et al. 2008). Based on data from Pellegrini et al. [32], the BF had a higher TEAC (~3.4 mmol/L) than several whole vegetables, including tomatoes, radish, potato, onion, lettuce, leek, green beans, artichokes, avocado, broccoli, cabbage, carrot and cauliflower. Interestingly, the TEAC of a number of commonly consumed fruits, such as apples (~1.45 mmol/L), bananas and pears, were also found to be lower than the BF [32]. However, as seen with the BTJ, berried fruits such as blackberry, strawberry and raspberry exhibited a significantly higher AC than BF. Furthermore, the BF had a lower AC than the reported values for fresh beetroot extracts (~3.4 vs. ~5.21 mmol/L) but similar to a cooked beetroot extract (~2.94 mmol/L) [32]. This suggests that some of the active compounds in beetroot are lost or perhaps degraded during cooking and processing. Conceptually, thermal treatment, exposure to bacterial agents, acidification, storage conditions, and modified atmospheric treatment could all affect phytochemical composition [33, 34]. Despite this, the BF and BTJ in particular still possess reasonably high antioxidant capacities in comparison with other fruit and vegetables and may therefore be favourable sources for boosting antioxidant defences and protecting against conditions associated with oxidative damage.

The antioxidant activity of the BTJ and BF can probably be ascribed to the high concentration of polyphenols and betalains they contain (see Table 2) and also to any synergistic interactions that might occur with these compounds, as has been suggested previously [35]. As our main focus was on betanin, quantifying individual polyphenols in the BTJ and BF was beyond the scope of this study. However, according to data from previous investigations, the main polyphenols in beetroot are phenolic acids (ferulic acid, chlorogenic acid, caffeic acid) and flavonoids (epicatechin, rutin, betagarin) [29, 35], many of which possess high antioxidant potential [36, 37]. Furthermore, the polyphenols in beetroot appear to be well absorbed in humans. Netzel et al. [38] reported that 51 % of the total phenolics (about 338 mg) ingested from a homemade beetroot juice were detectable in the participants urine, indicating that several of the polyphenols present in beetroot may be absorbed and made available in the circulation for physiological effects.

Both the juice and food were rich in betalain compounds (Table 2). In accordance with studies on fresh beetroot extracts, the betaxanthin content of BTJ and BF was much lower than the betacyanin content [39]. Betacyanins appear to be stronger antioxidants than betaxanthins [10, 39] and were likely major contributors to the antioxidant activity demonstrated by the BTJ and BF. Interestingly, the betanin content of BTJ was much higher than BF (~194 mg vs. ~66 mg per serving). The reason for this is unclear, but is probably due to differences in how the products are processed or possibly the extraction methods used for analysis. Regardless, the higher TEAC values for BTJ versus BF are probably due to its comparatively higher betanin content. The antioxidant potential of betanin is believed to be higher than other betalains present in beetroot [10, 14, 17].

To our knowledge, this is the first study that has characterised the bioavailability of betanin in human plasma following beetroot consumption. Despite the relatively high amount of betanin present in both the BTJ and BF, it could not be identified in the plasma at any time point after consumption (1–8 h). Our findings conflict with those of a previous study, in which betanin was identified in plasma at relatively high concentrations (~0.2 μmol/l) 2 h after consuming 500 g of fresh cactus pear fruit containing 16 mg of betanin [40]. However, the discrepant findings between this study and the present investigation could be related to differences in the foods analysed (i.e. cactus pear fruit versus beetroot). This is supported by recent work from Tesoriare et al. [41] who compared the absorption rates of betanin from cactus pear fruit and red beetroot in a simulated in vitro model of the intestinal wall. They showed that epithelial transport was much lower when betanin was derived from red beetroot, speculating that the rate of absorption was inhibited by beetroot’s food matrix. This suggests that the bioavailability of betanin may be lower after beetroot consumption compared to other sources of betanin.

Nevertheless, our inability to identify betanin in the plasma suggests that it may be lost or degraded during digestive processes. Previous studies investigating the renal elimination of betalains have indicated that betanin may instead be absorbed as downstream metabolites [17]. Kanner et al. [17] found that after consuming a betanin-rich beetroot juice, isobetanin, but not betanin, could be detected in urine. The authors suggested that betanin undergoes isomerisation to isobetanin in the intestinal milieu and may therefore be the major metabolite absorbed after betanin ingestion. In addition to isomerisation, there are several other metabolic processes that could degrade betanin and limit its systemic bioavailability, including glycosidase enzyme activity from cellulase [17] or the presence of the pancreatic enzyme amylase [33]. Such data support the possibility that betanin is largely metabolised to secondary compounds prior to entering the circulation, which would provide a potential explanation as to why we were unable to detect betanin in the present study. This raises doubts as to whether the wide array of biological effects displayed by betanin in vitro can be extrapolated to in vivo conditions. Instead, the in vivo biological activity displayed by betanin in some studies [42, 43] could be mostly due to the biological effects of secondary betanin metabolites, although this remains to be elucidated. At present, data on the bioavailability of these metabolites or their potential biological activity are not yet available. Unfortunately, we were unable to unequivocally identify any metabolites of betanin in this study due to appropriate standards for HPLC/LCMS detection not being available. Thus, whether metabolites of betanin reached the circulation in the present study is purely speculative until clarified with future research. The development of new methodologies, analytical techniques and suitable standards will be required to establish the presence of these compounds.

Plasma NOx activity was significantly augmented after consumption of both BTJ and BF compared to the placebo (Fig. 3). These findings agree with a previous study that reported a rapid rise in NOx activity 1–3 h after BTJ ingestion [8]. Collectively these data are important, because an increase in the endogenous NOx pool is associated with a range of physiological effects that might be beneficial to health, such as improved endothelial function, reduced blood pressure, enhanced mitochondrial efficiency and improved metabolic function [5].

We acknowledge that a potential limitation of this study is the absence of any measures of urinary excretion. Therefore, we cannot rule out the possibility that betanin would have been detectable in excreted urine had we collected samples after beetroot consumption. Because the urinary elimination of betanin has been described before [17, 38], we chose to focus specifically on plasma bioavailability, which to the best of our knowledge, has not been characterised after beetroot consumption. We also acknowledge that restricting our analysis to 8 h post-consumption is a possible limitation because betanin might have appeared in the plasma at later time points. However, we feel that this is unlikely based on previous work in cactus pear fruit that showed plasma betanin concentration peaked at 3 h post-consumption and was undetectable at 8 h post [40]. Finally, our analysis focused exclusively on betanin and nitrate, and it was beyond the scope of this study to analyse individual phenolic and betalainic compounds present in beetroot. However, we acknowledge that these are not the only compounds in beetroot that have the potential to exert beneficial physiological effects. We therefore thought it prudent to describe the total phenolic, antioxidant capacity and betalainic content of these foods to demonstrate that there are in fact other phytonutrients in these foods. However, further research is required to delineate the individual bioactive compounds present in these particular foods.

Despite the aforementioned limitations, the data presented in this study provide new information on the bioavailability and phytochemical content of commercially available beetroot juice and whole food and will serve to stimulate further research into the effects of beetroot, while also being useful to practitioners interested in the potential health benefits of these products. Future research on the bioavailability of betanin metabolites and their potential for biological activity are required to further exude the potential usefulness of beetroot foods for health.
